# Influence Mechanism of Different Environmental Regulations on Carbon Emission Efficiency

**DOI:** 10.3390/ijerph192013385

**Published:** 2022-10-17

**Authors:** Liang Liu, Mengyue Li, Xiujuan Gong, Pan Jiang, Ruifeng Jin, Yuhan Zhang

**Affiliations:** School of Economics and Management, Southwest University of Science and Technology, Mianyang 621010, China

**Keywords:** environmental regulation, carbon emission efficiency, fuzzy-set qualitative comparative analysis (fsQCA), super slacks-based measure of efficiency (SE-SBM)

## Abstract

The rational use of environmental regulation tools has become an important means by which to improve the efficiency of carbon emissions. Different types of environmental regulations and their combinations have different impacts on carbon emission efficiency. In order to determine the environmental regulation configurations that may achieve high carbon emission efficiency or lead to low carbon emission efficiency, we constructed an analytical framework of connections between environmental regulation configurations and carbon emission efficiency. Moreover, 30 Chinese provinces from the period covering 2016 to 2019 were selected as research cases. In addition, the super slacks-based measure of efficiency (SE-SBM) model was applied to evaluate carbon emission efficiency. Finally, the fuzzy-set qualitative comparative analysis (fsQCA) method was employed to analyze the impact of different environmental regulation configurations on carbon emission efficiency. The results showed that the carbon emission efficiency of various regions of China is generally low (with most regions not having reached an effective level) and that there are large regional differences. We found that there are four environmental regulation configurations that can achieve high carbon emission efficiency and two environmental regulation configurations that lead to low carbon emission efficiency. Based on these configurations, we draw three conclusions: (1) There are three paths to achieving high carbon emission efficiency: one that values command-and-control environmental regulation but disfavors market-incentive environmental regulation, another that combines command-and-control environmental regulation with market-incentive environmental regulation, and a third that couples command-and-control environmental regulation with voluntary environmental regulation. (2) Two paths that may lead to low carbon emission efficiency were established: excessive penalties and the lack of specific measures. (3) In some conditions, environmental governance investment and fiscal expenditure could be substituted for each other; environmental protection administrative penalties and pollution charges are synchronized; environmental governance investment in the promotion of carbon emission efficiency is indispensable. Policies and suggestions on how the government can use environmental regulation tools to improve carbon emission efficiency are proposed from a general coordinative perspective in the final section of this paper.

## 1. Introduction

Climate change has a bearing on human society’s sustainability. The Intergovernmental Panel on Climate Change (IPCC) released a Summary for Policymakers in the Sixth Assessment Report [1], which explains that human activities have had a profound impact on nature, with increasing frequency and intensity of extreme weather caused by human activities and greenhouse gas (GHG) emissions accelerating global warming at an unprecedented rate. Only with immediate, rapid, and mass actions will the global temperature control target of 1.5 °C be achieved, indicating escalating conflicts between economic development and environmental issues. Thus, decreasing greenhouse gas emissions became a consensus and central issue of the international community [2]. Since 2007, China has surpassed the United States to become the world’s largest carbon emitter. Additionally, China is still in the process of industrialization and urbanization, the demand for fossil fuels still exists [3], and the continuous increase in carbon emissions has also put China under enormous pressure from the international community. These facts show that the contradiction between China’s economic development and the ecological environment is serious, and economic development urgently needs to transform to low-carbon development [4]. How to coordinate the relationship between economic development and environmental protection will be the focus of Chinese society for a long time in the future.

Carbon dioxide (CO_2_), as a representative greenhouse gas, is a top priority in energy conservation and emission reduction. China, as a responsible great power, shoulders its heavy load of reducing carbon dioxide emissions and protecting the ecological environment. In 2015, China announced at the 21st United Nations Climate Change Conference in Paris that it will achieve a 60–65% reduction in CO_2_ emissions per unit of Gross Domestic Product (GDP) by 2030 compared with 2005, which was also included as an indicator in the “Twelfth Five-Year Plan”. In September 2020, President Xi Jinping proclaimed at the United Nations General Assembly that China would strive to peak its CO_2_ emissions by 2030 and work towards carbon neutrality by 2060. However, China is still in the ranks of developing countries. Therefore, carbon emission reduction cannot be at the expense of economic development. Improving carbon emissions efficiency is considered a feasible and effective means by which developing countries such as China [5] can achieve a proper balance between carbon emissions and economic activities [6,7]. Enterprises can develop green and low-carbon technologies to reduce carbon emissions and improve carbon emissions efficiency [8]. Compared with corporate behavior, the government can use environmental regulation to intervene in market behavior. Environmental regulation can not only improve carbon emission efficiency, but can also promote low-carbon economic development [9,10,11]. Many scholars have carried out research on the relationship between environmental regulation and carbon emission efficiency. On the one hand, environmental regulation can improve carbon emission efficiency due to “forced reductions” [12]. Conversely, due to the “green paradox” [13], environmental regulation is not conducive to improving carbon emission efficiency. Additionally, there may also be a nonlinear relationship between environmental regulation and carbon emission efficiency, that is, the impact of environmental regulation on carbon emission efficiency is uncertain [14]. On the other hand, there are three types of environmental regulation: command-and-control, market based, and voluntary consciousness [15]. Most studies focus on the impact of a single type of environmental regulation on carbon emission efficiency, ignoring the complexity of the action mechanism of environmental regulation tools and the possible combined effects of environmental regulation combinations. Therefore, it is worth further exploring the impact mechanism of different types of environmental regulations on carbon emission efficiency from the perspective of configuration.

Based on the above analysis, we aimed to better study the impact mechanism of different configurations of three types of environmental regulations on carbon emission efficiency. First, we present our analysis of the spatial distribution of China’s carbon emission efficiency and the reasons for the differences. Second, we discuss our analysis of the environmental regulatory configurations that may achieve high carbon emission efficiency or lead to low carbon emission efficiency. Finally, we explore the relationship between different condition variables under specific conditions. The rest of the paper is structured as follows: Section 2 reviews the relevant literature; Section 3 is the study design; Section 4 reports the data results of this paper; Section 5 presents our analysis of the path to achieving high carbon emission efficiency and the path leading to low carbon emission efficiency, as well as the relationship between condition variables; and Section 6 summarizes the conclusions of this paper and makes policy recommendations.

## 2. Literature Review

Environmental regulation (ER), as a significant method used to solve environmental problems, refers to institutional arrangements adopting new concepts, systems, and policies to guide public behavior so as to save resources and protect the environment [16]. Within the basic type, there are three types of environmental regulations: command-and-control environmental regulation, market-incentive environmental regulation, and voluntary environmental regulation [15]. Command-and-control environmental regulation is characterized by its “coercion”, which guides public behavior with strict environmental regulations. Additionally, its specific regulatory methods include environmental regulation clauses [17], pollutant discharge standards [18], and cleaner production standards [15]. Market-incentive environmental regulation refers to market environmental management and controls with incentives, whose main administrative methods are green tax (pollution charges) [18], carbon emissions trading [15], and subsidies [17]. Voluntary environmental regulation consists of environmental protection commitments and voluntary actions made by the public. Its specific regulatory procedures mainly include voluntary participation [16], environmental protection information disclosure [15], and voluntary environmental agreements [19]. From the perspective of influencing factors, factors such as social interaction [20], technical efficiency level [21], and policy sustainability [22] also affect the implementation effect of environmental regulation.

As an environmental and economic concept, carbon emission efficiency (CEE) functions as an influential indicator for the estimation of both economic development and the effectiveness of energy conservation and emission reduction. Scholars have generally researched carbon emission efficiency according to measurement methods and contributory factors. Regarding the measurement method, in addition to Stochastic Frontier Analysis [23], academics principally adopt Data Envelopment Analysis (DEA) and its advanced models to conduct research from the aspect of measurement methods. For instance, the SE-SBM model was employed by Guo Pei [24] and Xie Zihan [25] to evaluate carbon emission efficiency in different countries and cities. Additionally, Undesirable-SBM [26], SE-SBM [27], SBM-DEA [28], and other models have played an important role in evaluating carbon emission efficiency in 30 Chinese provinces and cities. Moreover, carbon emission efficiency could be affected to different degrees by the following contributory factors: science and technology innovation [29], low-carbon city pilot policies [30], urbanization level, energy consumption structure (ECS), foreign trade, government intervention [31], and technological advances [25].

Research on links between environmental regulation and carbon emission efficiency expand largely on the “Porter hypothesis”. This hypothesis advocates that rigorous environmental regulation may stimulate enterprise innovation, which could offset environmental protection costs and produce enterprise benefits [32]. It is believed that environmental regulation has a positive impact on carbon emission efficiency in line with the “Porter hypothesis” [24].Taking the low-carbon pilot policy as an example, scholars have found through research that the policy has a positive effect on urban carbon emission efficiency [24] and industrial carbon emission efficiency [33]. However, some refuters of the hypothesis argue that environmental regulation will not improve carbon emission efficiency but may lead to reductions [13]. Moreover, scholars have taken different industries and regions as research objects, concluding that environmental regulation’s impact on carbon emission efficiency is uncertain [13,34].

Different types of environmental regulations have different effects on carbon emission efficiency. Some scholars believe that the carbon financial market, as a market-incentive environmental regulation tool, has a positive impact on improving carbon emission efficiency [11,35]. ZhenyuJiang divided command-and-control environmental regulations into industry regulations and regional regulations in order to conduct research. The results showed that mandatory environmental regulations had a certain impact on energy conservation and emission reduction [36]. Xiu Cheng’s analysis found that the use of voluntary environmental regulation to cultivate citizens’ awareness of environmental protection was more helpful in allowing citizens to generate positive low-carbon consumption behaviors [37]. The existing research mainly focuses on the implementation effects of different types of environmental regulations on carbon emission efficiency, and it has been found that the implementation effects vary significantly [35], but few studies have explored the comprehensive effects of different configurations of environmental regulation tools on carbon emission efficiency.

Although domestic and foreign scholars have made abundant achievements in environmental regulation and carbon emission efficiency, with some scholars even having explored environmental regulation’s impact on carbon emission efficiency, the research has some shortcomings: (1) the existing literature rarely considers diverse environmental regulations’ simultaneous impact on carbon emission efficiency while evaluating them, and (2) a single factor’s effects on carbon emission efficiency are discussed, but a configuration analysis of the contributory factors is seldom mentioned in studies on contributory factors of carbon emission efficiency. Based on these deficiencies, in this study, we carried out a fuzzy-set qualitative comparative analysis, and 30 Chinese provinces during a sample period from 2016 to 2019 were selected as the main research objects based on the energy saving and emission reduction requirements of the Paris Agreement in 2015 and the Twelfth Five-Year Plan. Command-and-control environmental regulation (environmental administrative penalties and environmental governance investment), market-incentive environmental regulation (pollution charges and fiscal expenditure), and voluntary environmental regulation (suggestions and proposals) were taken as the antecedent conditions for environmental regulation theory, externality theory, and low-carbon economy theory. Moreover, the carbon emission efficiencies calculated by the undesirable output SE-SBM model were regarded as the consequences. Furthermore, the configurations of the antecedent variables that affect carbon emission efficiency in various regions were identified and explained by investigating causal relations between different conditions and consequences so as to explore the impact of different environmental regulations on carbon emission efficiency and to provide targeted suggestions for improving carbon emission efficiency in different regions.

## 3. Research Design

### 3.1. Analytical Framework

Environmental regulation refers to governmental policy-making and measures implemented to guide public behavior towards protecting the environment. Externality theory is used to better understand the costs or benefits of economic activities experienced by unrelated third parties [38]. Environmental pollution presents a strong negative externality. Polluters will not stop harming the environment if the cost borne by them is far lower than that by society. On the contrary, environmental protection has a positive externality, but the benefits for environmental protectors are far lower than those for society. Hence, protectors lack the motivation to provide the environmental protections that society needs. Therefore, it is necessary to rely on environmental policies to reduce pollution and encourage protection. Accordingly, whether different environmental regulation designs are reasonable relates to the successful implementation of environmental policies [39].

The low-carbon economy is supposed to achieve economic development and eco-environmental protection coordinately through technology, concepts, and industrial structure innovation to reduce carbon emissions [40]. Carbon emission efficiency, whose basis of manifestation is the low-carbon economy theory, lays stress on output effects triggered by carbon emissions. Furthermore, it is the carbon emission quantity that limits carbon emission efficiency, and economic growth that promotes it. Environmental regulation, as a policy measure, imposes a “reverse transmission” or a “retrogressive effect”(the green paradox [41]) on carbon emission efficiency. That is to say, the former argues that an increase in the intensity of environmental regulation encourages low-carbon technological innovations in enterprises, which in turn improves resource utilization and promotes economic advances. The latter argues that an increase in the intensity of environmental regulation will offset the positive effects of environmental protection due to increased costs, thereby inhibiting carbon emission efficiency, which demonstrates that the study of environmental regulations’ impacts on carbon emission efficiency is consistent with low-carbon economy theory [42].

**Figure 1:**
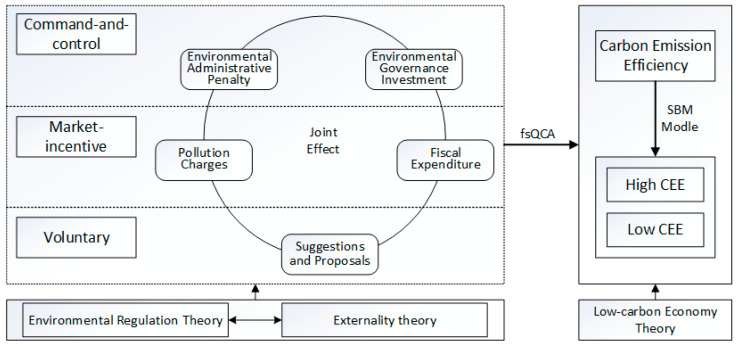
The analytical framework of environmental regulation configurations.

Therefore, we selected different environmental regulations as antecedent variables and carbon emission efficiency as consequences based upon environmental regulations, externality theory, and low-carbon economy theory to explore how various environmental regulations can be configured so as to promote carbon emission efficiency and to analyze which configurations cause carbon emission inefficiency. The analytical framework of the environmental regulation configurations is shown in Figure 1, drawn using Microsoft Visio software (Microsoft, Redmond, America).

According to the relevant research on environmental regulation, we adopted three types of environmental regulations: command-and-control environmental regulation, market-incentive environmental regulation, and voluntary environmental regulation [43]. Command-and-control environmental regulation refers to governmental compulsory penalties on polluters enacted through policies and regulations, which has strong coercion [44,45]. However, some polluters ignore environmental pollution consequences to maximize their profits [46]. Only when the loss due to strong penalties is greater than their expected incomes will they take measures to promote carbon emission reduction and environmental governance [47]. Accordingly, the intensity of command-and-control environmental regulation affects carbon emission efficiency. We evaluated command-and-control environmental regulation according to environmental administrative penalties and environmental governance investment. Market-incentive environmental regulation guides polluters through the market [48]. Moreover, the government provides incentives using positive externalities such as ensuring environmental protection by fiscal expenditures or collecting environmental protection taxes and pollution charges for negative externalities such as environmental pollution [49]. Market-incentive environmental regulation forces polluters to consider the associated environmental costs and to reform their production processes, which in turn, influence carbon emission efficiency. Measures such as pollution charges and fiscal expenditure were selected to assess market-incentive environmental regulation in this study. Voluntary environmental regulation builds upon public voluntary participation in environmental protection through environmental petition letters, environmental incident disclosure, and other means [50,51]. Additionally, the National People’s Congress (NPC) deputies, Chinese People’s Political Consultative Conference (CPPCC) members, and the general public put forward proposals on environmental issues, prompting the government and polluters to take relevant measures to improve carbon emission efficiency. In this study, suggestions and proposals were selected as measurements by which to evaluate voluntary environmental regulation.

### 3.2. Research Methodology

Firstly, we constructed an SE-SBM model of undesirable outputs, which builds upon the low-carbon economy theory to estimate carbon emission efficiency. Secondly, the fsQCA method was introduced, and its applicability to this study is illustrated.

#### 3.2.1. Super Slacks-Based Measure of Efficiency Model of Undesirable Output

DEA [52] is a method for evaluating the efficiency of decision-making units with multiple input–output quotas. Nevertheless, the traditional DEA model is premised on radial measurement, which assumes that inputs and outputs change in the same proportion, making the calculations unable to accurately explain actual situations. Based on this, Tone [53] proposed a non-angular and non-radial slack variable-based efficiency measurement method (Slacks-Based Measure), or the SBM model, in 2001, which, however, measured efficiency values all within the (0, 1] interval. It is impossible to compare efficient decision-making units. Accordingly, an SE-SBM model that is based on modified slack variables was constructed by Tone [54] in 2002, which can be used to evaluate decision-making units with efficiency values greater than 1 to achieve more accurate calculation results. With environmental issues receiving global attention, producers consider not only economically desirable outputs but also undesirable output constraints (carbon emissions) in pursuing low-carbon economic development. Therefore, we established an SE-SBM model that considers undesirable outputs to estimate CEE using the following equation:

(1) $$\rho =\min\frac{1+\frac{1}{m}\sum_{i=1}^{m}\frac{s_{i}^{x}}{x_{i0}}}{1-\frac{1}{s_{1}+s_{2}}\left(\sum_{k=1}^{s_{1}}\frac{s_{k}^{y}}{y_{k0}}+\sum_{l=1}^{s_{2}}\frac{s_{l}^{z}}{z_{l0}}\right)}\;s.t.\;x_{i0}\geq \sum_{j=1,\neq 0}^{n}\lambda_{j}x_{j}-s_{i}^{x},\forall i;\;y_{k0}\leq \sum_{j=1,\neq 0}^{n}\lambda_{j}y_{j}+s_{k}^{y},\forall k;\;z_{l0}\geq \sum_{j=1,\neq 0}^{n}\lambda_{j}z_{j}-s_{l}^{z},\forall l;\;1-\frac{1}{s_{1}+s_{2}}\left(\sum_{k=1}^{s_{1}}\frac{s_{k}^{y}}{y_{k0}}+\sum_{l=1}^{s_{2}}\frac{s_{l}^{z}}{z_{l0}}\right)>0;\;s_{i}^{x}\geq 0,s_{k}^{y}\geq 0,s_{l}^{z}\geq 0,\lambda_{i}\geq 0,\forall i,j,k,l.$$

where “n” represents the number of decision-making units. “m”, “$s_{1}$”, and “$s_{2}$” denote the number of inputs, desirable outputs, and undesirable output indicators, respectively. Meanwhile, $s_{i}^{x}$, $s_{k}^{y}$, and $s_{l}^{z}$ signify the corresponding slack variables and $\lambda_{i}$ represents the weight vector.

#### 3.2.2. Fuzzy-Set Qualitative Comparative Analysis

Qualitative Comparative Analysis (QCA) [55] is a set-theoretic research method based on Boolean operations that can be used to explore causal relations between different configurations of conditional variables and consequences, finding combinations of antecedent conditions that lead to the occurrence or non-occurrence of an outcome [56].

The robustness of the results provided by the QCA method depends on the inclusion of representative individuals, rather than on the sample size. Therefore, the method applies to small and medium-sized samples. Moreover, clear-set qualitative comparative analysis (csQCA) and multi-valued qualitative comparative analysis (mvQCA) operate on categorical variables, while fuzzy-set qualitative comparative analysis (fsQCA) [57] solves degree variations and partial membership issues. In fsQCA, variables are assigned values through membership value calibration and consistency assessment to more precisely reveal the causal relations between conditional variable configurations and consequences. Calibration means a variable’s conversion to a fuzzy value, specifically the use of a logistic function to fit the original data to full membership values, cross-over points, and full non-membership values, which can be used to determine whether the condition belongs to the set [58]. One needs to analyze the necessity of single variable before using fsQCA for configuration analysis. Generally, the consistency of a single variable is used to determine whether it is a necessary condition, that is to say, if the consistency value of the conditional variable is over 0.9, then it is a necessary condition for the result; thus, this condition variable should be eliminated [59]. Consistency and coverage are two important indicators used to interpret the results of multiple combinations [60]. The consistency metric, whose value is in the range of 0 to 1, usually more than 0.8, measures whether the condition configurations form a subset. The coverage metric indicates degrees of interpretation of the results caused by the configurations of different conditional variables and also has a value in the range of 0 to 1.

We applied the fsQCA method to analyze how synergistic effects of different environmental regulations in China affect carbon emission efficiency for the following main reasons. Firstly, carbon emissions efficiency is influenced by different environmental regulations This method can be used to examine the configuration effects of diverse environmental regulations on carbon emission efficiency from a holistic perspective. Secondly, Chinese provinces differ in their development levels and factors. Thus, it is necessary to adjust measures to local conditions to provide carbon emission efficiency improvement paths. Moreover, this method can condense multiple equivalent paths in a targeted manner. Finally, the methodology is also applicable to small and medium-sized samples. We took 30 Chinese provinces, autonomous regions, and municipalities as samples, which satisfies the requirements of fsQCA being applicable to small and medium-sized samples.

### 3.3. Variable Design

#### 3.3.1. Consequences

We selected average values for the carbon emission efficiency (ACEE) of each province from the years 2016 to 2019 as the consequence based on previous analyses, which were estimated using MATLAB software to build an SE-SBM model of undesirable output. Moreover, when calculating the carbon emission efficiency index from 2016 to 2019, we selected the employment rate at the end of the years in each province as the labor inputs. Additionally, we calculated the fixed capital stock of each province using the perpetual inventory method [61] to serve as the capital inputs, with the depreciation of fixed capital stocks at a rate of 10.96%. The total energy consumption of each province was selected as the energy input. Moreover, the desirable output was expressed as regional GDP, and the year 2016 was selected as the base period for calculation and adjustments to the real GDP. The undesirable output was expressed as carbon dioxide emissions, which is the sum of carbon dioxide emissions from various energy sources. The definitions of the input–output variables are detailed in Table 1.

#### 3.3.2. Conditional Variables

Five indicators were selected as the conditional variables from the three environmental regulations in this study, namely, command-and-control environmental regulation measured by environmental administrative penalties (EAP), and environmental governance investment (EGI). Market-incentive environmental regulation was estimated according to pollution charges (PC) and fiscal expenditure (FE), and voluntary environmental regulation was evaluated by suggestions and proposals (SP), as detailed in Table 2.

EAP has three main functions: legal deterrence, risk prevention, and ecosystem restoration, by penalizing producers for environmental violations [62,63] and requiring them to regulate their production, thus affecting carbon emission efficiency. The average number of environmental administrative penalty cases in each province from 2016 to 2019 was selected as a measurement.

EGI [64] refers to governmental fiscal funds for environmental pollution control. As conflicts between economic development and environmental protection grow, governments are strengthening their regulations to improve environmental quality through investment in environmental pollution control, which, in turn, affects carbon emission efficiency in provinces. The average amount of environmental pollution control investment as a proportion of the regional GDP of each province from 2016 to 2019 was selected as a measurement.

PC [65] means that producers must pay discharges for environmental pollution during production, which, on the one hand, increases enterprises’ costs and reduces their investment in green technology innovation, thus affecting carbon emissions efficiency [66]; however, on the one hand, this may have a “reverse transmission” effect, helping producers to innovate in green technologies, which not only leads to energy saving and emission reductions, but also allows them to capture market share and promote economic growth due to their gaining competitive advantages. The average share of the pollution charge revenue in the regional GDP of each province from 2016 to 2019 was selected for assessment.

Fiscal expenditure on environmental protection is designed to promote the harmonization of economic development and environmental protection, which is a positive incentive for environmentally protective behavior [67]. Pearce and Palmer [68] argue that environmental fiscal spending should theoretically be related to environmental demand effects and gross national product. Furthermore, this could influence economic growth and environmental quality, which, in turn, has an impact on carbon emission efficiency. The average share of fiscal expenditure on environmental protection in the regional GDP of each province from 2016 to 2019 was chosen to characterize this indicator.

SP [69] includes NPC suggestions and CPPCC proposals, i.e., suggestions and proposals made by NPC deputies and CPPCC members in response to environmental issues, which are supposed to be answered by the authorities and reported by the media. Producers will consciously implement energy saving and emission reduction measure to maintain their environmental credibility and brand awareness, thus affecting carbon emission efficiency. The average sum of the number of NPC suggestions and CPPCC proposals from 2016 to 2019 in each province was selected to characterize this indicator.

### 3.4. Case Selection

The fsQCA method is suitable for small and medium-sized samples and can be used to investigate the configuration effects of multiple antecedents by combining different representative cases. We studied the contribution paths of distinct environmental regulation configurations in Chinese provinces on carbon emission efficiency. Accordingly, 30 provinces, municipalities, and autonomous regions in China (the Tibet, Hong Kong, Macao, and Taiwan regions were excluded considering data availability limitations) were selected as research samples.

## 4. Results

### 4.1. Result of Slacks-Based Measure

**Figure 2:**
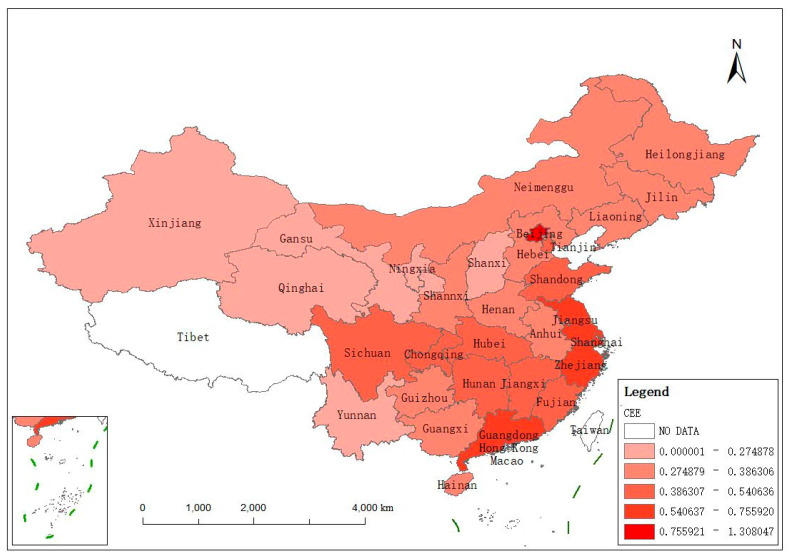
The average carbon emission efficiency (ACEE) in different Chinese provinces from 2016 to 2019.

The carbon emission efficiency of 30 Chinese provinces was calculated based on Formula (1). For a more visual representation, the mean values of the carbon emission efficiency of these provinces from 2016 to 2019 are depicted in Figure 2, creating using the ArcGIS software (ESRI, RedLands, CA, USA). According to Figure 2, the efficiency of the 30 provinces differed from each other during the sample period. The efficiency was low in most areas of China, and the values of efficiency in Beijing, Jiangsu, Shanghai, Zhejiang, and Guangdong were higher than 0.5406. The reason for this may be that these regions are able to rapidly respond to ecological policies and have abundant capital, pools of talent, and technical resources. The values of efficiency in Yangtze River Basin region, such as Sichuan, Chongqing, Hubei, Hunan, and Jiangxi, were from 0.3863 to 0.5406. The values in the rest of the areas were lower than 0.3863; these regions are rich in resources but inefficient in utilization and limited in economic development; thus, their low carbon emission efficiency also tallies with reality.

**Figure 3:**
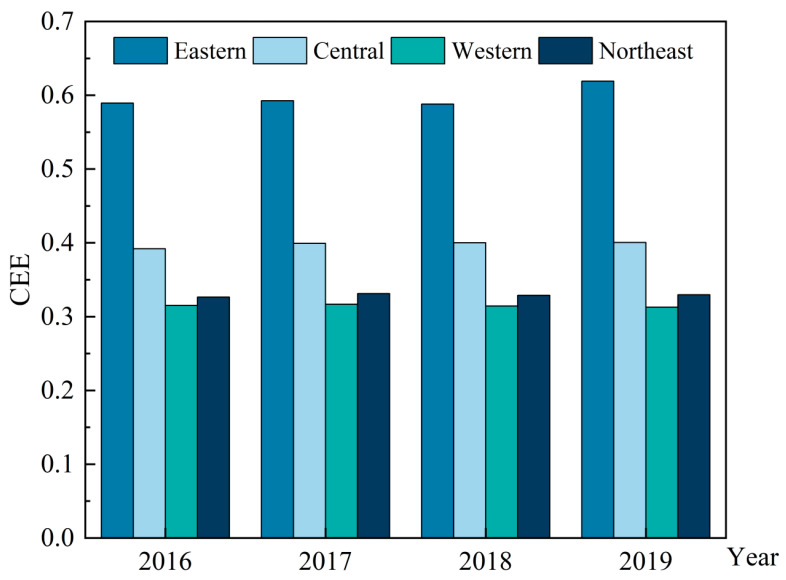
Comparison of the average carbon emission efficiency (ACEE) of the four economic regions in China from 2016 to 2019.

To further analyze the differences and variations in carbon emission efficiency, we divided China into eastern, central, western, and northeastern regions in this study according to the relevant policies. The carbon emission efficiency of provinces from 2016 to 2019 is shown in Figure 3, which was drawn using Origin software(Electronic Arts, Redwood City, CA, USA). Similar to the result reported in Figure 2, carbon emission efficiency can be seen to gradually decrease in the eastern central, northeastern, and western regions from 2016 to 2019 [70,71,72,73]. The efficiency value in the eastern region was around 0.6, which is a high. With more interaction with the outside world, the eastern coastal areas are abundant in talent and capital resources, and they can gain timely access to cutting-edge green technologies to promote the transformation and upgrading to the tertiary industry. Therefore, the carbon emission efficiency in these areas was much higher than that of others. The carbon emission efficiency value of the central region was around 0.4, much lower than the eastern region. The central region has undertaken the industrial transfer from the eastern region, with a relatively high share of industry and abundant energy resources, but the levels of economic development and utilization of technology in this area prevent it from achieving efficient energy utilization. Moreover, the values of efficiency in the northeastern and western regions were around 0.3, which are much lower than those in the eastern region, while the northeastern carbon emission efficiency was slightly higher than that of the western region. Due to slow economic restructuring, serious brain drain, and resource exhaustion, the northeastern region lags behind the eastern and central regions in carbon emission efficiency. Located in the hinterland of China, the western region is limited in economic and technological development because of its geographical location. Therefore, this area could not achieve reasonable mining, resulting in low carbon emission efficiency.

### 4.2. Analysis of Efficiency Contribution Path

#### 4.2.1. Descriptive Statistical Analysis

The descriptive statistical analysis of each variable is shown in Table 3, which demonstrates that the standard deviation of the ACEE variables was 0.2117, the minimum was 0.2094, and the maximum was 1.3080. This shows that there are major differences between the carbon emission efficiency values in different provinces of China. Additionally, there were also significant differences in each conditional variable, which contributed to our exploration of the influence of different environmental regulations on carbon emission efficiency. Table 4 reports the multicollinearity test for each variable. It can be seen from the table that the VIF value of each variable was less than 3, and that the mean VIF value was 1.96. Therefore, there was no collinearity problem between the variables.

#### 4.2.2. Variable Calibration

Variable calibration is a crucial step in fsQCA, and the threshold should be set according to actual situations and data features [58]. We drew on existing documents, and took 75%, 50%, and 25% quantiles of the raw data of five conditional variables and one consequence as full membership, cross-over points, and full non-membership, respectively. The selection of the thresholds for each variable is shown in Table 5.

#### 4.2.3. Analysis of Necessary Conditions

Table 6 presents the necessity analysis of a single variable. From this, it can be seen that the consistency values of all the single variables were below 0.9, suggesting that none of the conditional variables would lead to results on their own. Therefore, it is feasible to discuss the influences of different environmental regulation configurations on carbon emission efficiency.

#### 4.2.4. Analysis of Conditional Configuration

An adequacy analysis mainly analyzes the adequacy of the configurations formed by different antecedent conditions regarding the result, and it is used to explore the antecedent conditions or the combination of variables that lead to the results. The consistency value should not be lower than 0.8 in most studies; thus, the consistency threshold of each configuration was set as 0.8 in this study. Meanwhile, there were only 30 cases included in the research, and then the frequency value is set as 1. The complex, intermediate, and parsimonious solution can be obtained from the analysis by using fsQCA 3.0. From the analysis of the intermediate and parsimonious elements, as shown in Appendix A Table A1 and Table A2, if there are intermediate and parsimonious elements in variables, then these variables are the core elements [74]. If there is only intermediate elements, these variable are the peripheral elements. Table 7 shows the configuration analysis results of different environmental regulations on high and low carbon emission efficiency.

As shown in Table 7, three antecedent configurations led to high carbon emission efficiencies, while two lead to low carbon emission efficiencies. The consistency indicates the extent to which each configuration solution and each overall configuration solution are a subset of the results [57]. Their values were 0.9002 and 0.8444, respectively, both higher than the theoretical value of 0.8, suggesting that each configuration is the necessary condition for the result. The coverage represents the degree to which the configuration solution can explain the results [57]. The overall coverages of the configuration solution value of the two results were 0.3150 and 0.4455, respectively, indicating that each configuration can reasonably explain the results.

Configurations 1 to 3 were the environmental regulation configurations that led to high carbon emission efficiency. The values of consistency and raw coverage for configuration 1 (EAP*EGI*~PC*~FE*~SP) were 0.8473 and 0.0745, respectively, in which the presence of EGI and the absence of PC are core elements, and the presence of EAP, the absence of FE, and SP are peripheral elements. The values of consistency and raw coverage for configuration 2 (EAP*~EGI*~PC*FE*~SP) were 0.8808 and 0.0893, respectively, in which the presence of EAP and FE and the absence of EGI and PC are core elements, and the absence of SP is a peripheral element. The values of consistency for configuration 3 (~EAP*EGI*~PC*~FE*SP or EAP*EGI*PC*~FE*SP) were 0.8889 and 0.8562 and the values of raw coverage were 0.1021 and 0.1719. The presence of EGI and SP and the absence of FE are core elements. The absence of EAP and PC are peripheral elements in 3a, while the presence of EAP and PC are peripheral elements in 3b. Configurations 4 and 5 were environmental configurations that led to low carbon emission efficiency. The values of consistency and raw coverage for configuration 4 (EAP*EGI*PC*~SP) were 0.8814 and 0.1377, in which the presence of EAP and PC and the absence of SP are core elements, while the presence of EGI is a peripheral element. The values of consistency and raw coverage for configuration 5 (~EAP*~EGI*~PC*~FE*~SP) were 0.8371 and 0.3435, in which the absence of EAP, EGI, FE, and SP are core elements, while the absence of PC is a peripheral element.

## 5. Discussion

### 5.1. Three Paths to Achieving High Carbon Emission Efficiency

Scholars have studied the role of each type of environmental regulation in promoting carbon emission efficiency [75,76], and this study focused on exploring how different types of environmental regulations can be combined to achieve high carbon emission efficiency, providing ideas for provinces to improve environmental policies. Three paths to achieving high carbon emission efficiency can be concluded from the core conditions of the four configurations, as shown in Table 7. The three paths are described as follows.

Configuration 1 is a path that encourages command-and-control environmental regulation rather than market-incentive environmental regulation. High carbon emission efficiency can be achieved in this path by using command-and-control environmental regulation while reducing market-incentive environmental regulation and voluntary environmental regulation, whose core condition is to apply environmental governance investment efficiently in command-and-control environmental regulation. Dong [77] found that, the greater the investment in environmental pollution control, the better the environmental quality. Similarly, we confirmed that with the increase in investment in environmental pollution control, environmental quality was be improved, thereby enhancing carbon emission efficiency. Consistent with the conclusions of Petroni’s [66] research, a core element is that pollution charges are not used as a tool of environmental regulation because these lead to increases in production costs; moreover, higher pollution charges can reduce economic output, thus leading to inefficient carbon emissions. Therefore, in contrast to other studies [66,77], we propose, from a configuration perspective, that when there is a lack of public voluntary environmental consciousness, provinces that strengthen investment in environmental pollution control while significantly reducing pollution charges, increasing administrative penalties according to the merits of each case, and reducing fiscal subsidies as far as possible can achieve high carbon emission efficiency.

Configuration 2 is a path that combines command-and-control environmental regulation and market-incentive environmental regulation. Environmental administrative penalties and fiscal expenditure are its core elements, in which the former represents command-and-control environmental regulation and the latter stands for market-incentive environmental regulation. According to the related research on the presence of two core variables [62,67], the deterrent effect of strong environmental administrative penalties and advanced fiscal support for environmentally friendly behaviors can encourage producers to pursue cleaner production and green technology innovation, thereby improving their carbon emission efficiency. Another core element is zero environmental governance investment and pollution charges, in which the former belongs to command-and-control environmental regulation and the latter is a part of market-incentive environmental regulation. According to the “green paradox” [41], strengthened environmental governance investment and pollution charges increase environmental costs, thereby accelerating resource consumption and increasing carbon emissions during a certain period, which results in few improvements in carbon emission efficiency. As a consequence, previous studies have only discussed the impact of individual environmental regulations on carbon efficiency [41,62,67], but this study draws conclusions from a configuration perspective. When there is a lack of public voluntary environmental consciousness, provinces that strengthen administrative penalties and fiscal expenditure and reduce their pollution charges as part of environmental governance investment can achieve high carbon emission efficiency.

Configurations 3a and 3b, two configuration solutions coming from the same parsimonious elements, are paths that combine command-and-control environmental regulation and voluntary environmental regulation, in which submitting proposals on environmental issues is the core element. The proposals and suggestions of the NPC and CPPCC reflect the intensity of public voluntary consciousness of environmentally friendly behaviors, and a higher voluntary consciousness could supervise and constrain production to improve carbon emission efficiency. This view confirms Jin’s view [69]. Environmental governance investment and the lack of financial support for environmental protection are core elements. Environmental governance investment can make up for the lack of environmental fiscal expenditure and improve environmental quality. As penalties for the negative externalities of environmental pollution, environmental administrative penalties and pollution charges are peripheral elements. These only play an auxiliary role in this path regardless of whether they exist or not. As a result, we propose, from a configuration perspective, that, with a strong public voluntary environmental consciousness, provinces that increase their environmental governance investment while reducing fiscal expenditure will achieve high carbon emission efficiency.

### 5.2. Two Paths That Cause Low Carbon Emission Efficiency

Similarly, some scholars have also studied the inhibitory effect of different types of environmental regulations on carbon emission efficiency [13]. In this study, we focused on investigating the kinds of configurations of different types of environmental regulations lead to low carbon emission efficiency from the perspective of configuration, thereby providing a warning for provinces. Two paths of low carbon emission efficiency can be concluded from the core elements of two configurations shown in Table 7. These are excessive penalties (configuration 4) and no measures (configuration 5).

Configuration 4 is a path of excessive penalties, in which the use of administrative penalties and pollution charges are core elements because both are penalties for the negative externalities of environmental pollution. Consistent with Ma’s findings [78], a high penalty increases environmental protection costs for producers, resulting in insufficient green innovation impetus, a reduction in economic output, and low carbon emission efficiency. Therefore, when there is a lack of strong public voluntary environmental consciousness, provinces that adopt high environmental administrative penalties and pollution charges have inefficient carbon emissions.

Configuration 5 is a path that lacks measures, i.e., a path without the three environmental regulations. More specifically, the lack of environmental administrative penalties, environmental governance investment, pollution charges, and financial support cause producers to lose motivation to develop clean resources and green technology innovation. As a consequence, carbon emission efficiency is insufficient in provinces that make no use of any of the environmental regulation tools to restrain pollution when there is a lack of strong public voluntary environmental consciousness.

### 5.3. Relationship among Conditions

After analyzing the conditions’ configurations, the interaction between elements can be understood through a horizontal comparison of the relations between each configuration solution.

Under certain conditions, environmental governance investment and fiscal expenditure can be substituted for each other. It was found by comparing configuration 1 and configuration 2 that EGI and FE cannot coexist with the presence of EAP and the absence of PC and SP. In other words, the presence of EAP and EGI and the absence of PC, SP, and FE can lead to high carbon emission efficiency, as can the presence of EAP and FE and the absence of PC, SP, and EGI.

In the given conditions, environmental administrative penalties and pollution charges are synchronous. By comparing configurations 3a and 3b, it can be seen that it is the simultaneous presence or absence of EAP and PC that leads to high carbon emission efficiency under the presence of EGI and SP but with the absence of FE.

As a core element, the presence of EGI appears three times in configuration that achieved high carbon emission efficiency. The absence of EGI is a core element in configuration 5, which achieved low carbon emission efficiency, indicating its relative importance in achieving high carbon emission efficiency [64].

## 6. Conclusions and Suggestions

Based on the relevant data from 30 Chinese provinces from 2016 to 2019, carbon emission efficiencies were calculated by establishing an SE-SBM model, and fsQCA was applied to study the contribution paths of different environmental regulation configurations on carbon emission efficiency. The conclusions are as follows:

Firstly, carbon emission efficiency is generally low across the Chinese provinces Most regions do not reach an effective efficiency level, and there are significant gaps among provinces.

Secondly, no single environmental regulation can act as the necessary condition for carbon emission efficiency. On the one hand, there are three paths to achieving high carbon emission efficiency: one that encourages command-and-control environmental regulation rather than market-incentive environmental regulation, another that combines command-and-control environmental regulation with market-incentive environmental regulation, and a third that combines command-and-control environmental regulation and voluntary environmental regulation. On the other hand, two paths that may lead to low-carbon emission efficiency are established: excessive penalties and the lack of specific measures.

Thirdly, under certain conditions, investment in environmental governance and fiscal expenditure can be substituted for each other. Moreover, the role of investments in environmental governance cannot be underestimated in improving carbon emission efficiency.

Based on the above findings, we put forward the following suggestions:

Firstly, the central government ought to improve the overall national carbon emission efficiency and narrow the gap between the eastern region and the other regions. It is necessary to pay attention to the diffusion of resources, such as energy-saving and emission-reduction technology, talent, and capital, from the eastern region to the other regions. The central government should lead the transition from a high-polluting energy industries to technology-intensive industries in the central, western, and northeastern regions. Efficient utilization of energy should be guaranteed while also achieving sustained economic growth, thus realizing a national improvement in carbon emission efficiency.

Secondly, local governments need to emphasize the adaption of various environmental regulations according to local conditions. They are expected to give full consideration to the local conditions and relations among different environmental regulations to avoid blindly blending different environmental regulations or adopting a single regulation, by which an overall high regional carbon emission efficiency could be achieved. Additionally, more attention should be paid to market-incentive and voluntary environmental regulations. The current environmental regulations based on command-and-control environmental regulation should be transformed into predominantly market-incentive and voluntary environmental regulations, which would allow for voluntary improvements in carbon emission efficiency in the relevant regions.

Thirdly, a low-carbon economic system should be established under governmental guidance, with producers as the main body and the public as active participants. At present, the Chinese government uses three groups of environmental regulations to achieve high carbon emission efficiency: predominantly, command-and-control environmental regulation; a combination of command-and-control environmental regulation and market-incentive environmental regulation; and a combination of command-and-control environmental regulation and voluntary environmental regulation. However, there is still a lack of collaboration among the three types of environmental regulation. The government should work towards the improvement of the relevant systems to encourage producers and the public to become integrated into the construction of low-carbon economic systems, as well as to encourage or force producers to pursue cleaner production and green technology innovation to improve carbon emission efficiency.

This paper has discussed environmental regulation configurations that influence carbon emission efficiency, but there are several insufficiencies, as follows: firstly, there are other forms of environmental regulations that were not included in the configuration analysis due to data availability limitations. Secondly, we simply analyzed the effects of environmental regulations on carbon emission efficiency from a static perspective since fsQCA is suitable for cross-sectional data studies. Future studies are expected to further analyze the influences of other forms of environmental regulations on carbon emission efficiency and the time effects between the two.

## Figures and Tables

**Table 1:** Relevant variables of the inputs–outputs.

Names of Variables		Definitions of Variables		Measurement Units	Sources
Inputs	Labor input	Year-end total employment		Per ten thousand person	CSMAR
	Capital input	$K_{it}=K_{i,t-1}(1-\delta_{it})+I_{it}$	(2)	Hundred million	*Statistical Yearbook of China*
	Energy input	Total energy consumption according to standard coal consumption		Thousands of tons	*China Energy Statistics Yearbook*
Desirable output	Regional GDP	Real GDP adjusted for the 2016 base period		Hundred million	CSMAR
Undesirable output	CO^2^ emission amount	Sum of CO^2^ emissions from all energy sources		Thousands of tons	CSMAR

Notes: refers to the China Stock Market & Accounting Research Database. In Equation (2), *K_it_* denotes the fixed capital stock of provinces, and *I_it_* represents the amount of fixed capital investment in provinces in period *t*. $\delta_{it}$ stands for the depreciation rate.

**Table 2:** Definitions and sources of the variables.

Names of Variables		Abbreviation	Definitions of Variables	Measurement Units	Sources
Conditional variables	Environmental administrative penalties	EAP	The average number of environmental administrative penalty cases from 2016 to 2019	Piece	*China Environment Yearbook*
	Environmental governance investment	EGI	The average proportion of environmental pollution control investment in the regional GDP from 2016 to 2019	%	CSMAR
	Pollution charges	PC	The average proportion of pollution charges in the regional GDP from 2016 to 2019	%	*China Environment Yearbook*
	Fiscal expenditure	FE	The average proportion of fiscal expenditure on environmental protection in the regional GDP from 2016 to 2019	%	CSMAR
	Suggestions and proposals	SP	Mean value of the sum of National People’s Congress suggestions and CPPCC proposals from 2016 to 2019	Piece	*China Environment Yearbook*
Consequences	Average carbon emission efficiency	ACEE	Mean value of CEE in 2016-2019	-	-

Notes: CSMAR refers to the China Stock Market & Accounting Research Database.

**Table 3:** Descriptive statistical analysis of each variable.

Variable	Mean	Std. Dev.	Min	Max
ACEE	0.4270	0.2117	0.2094	1.3080
EAP	1672.50	2029.79	9	8982.25
EGI	0.0138	0.0119	0.0013	0.0497
PC	0.000399	0.000312	0.000048	0.001164
FE	0.0083	0.0017	0.0057	0.0116
SP	545.33	363.91	84.75	1255.75

Notes: The variables’ abbreviations come from the definitions and sources of the variables (Table 2).

**Table 4:** The multicollinearity test for each variable.

Variable	VIF	1/VIF
EAP	1.50	0.6653
EGI	2.50	0.3998
PC	2.54	0.3943
FE	1.33	0.7512
SP	1.93	0.5185
Mean VIF	1.96	

Notes: The variables’ abbreviations come from the definitions and sources of the variables (Table 2).

**Table 5:** Variable calibration thresholds.

Variable	Full Membership	Cross-Over Point	Full Non-Membership
ACEE	0.4955	0.3740	0.3078
EAP	2296.88	883.50	413.56
EGI	0.019420	0.010684	0.004994
PC	0.000543	0.000300	0.000135
FE	0.009638	0.008517	0.006759
SP	879.44	552.75	190.94

Notes: The variables’ abbreviations come from the definitions and sources of the variables (Table 2).

**Table 6:** Necessity analysis of single factors.

Variable	High ACEE		Low ACEE	
	Consistency	Coverage	Consistency	Coverage
EAP	0.682	0.687	0.438	0.447
~EAP	0.451	0.442	0.693	0.689
EGI	0.602	0.623	0.435	0.457
~EGI	0.475	0.454	0.641	0.620
PC	0.508	0.498	0.559	0.556
~PC	0.547	0.550	0.495	0.505
FE	0.533	0.546	0.522	0.542
~FE	0.553	0.532	0.563	0.550
SP	0.630	0.657	0.398	0.422
~SP	0.445	0.422	0.676	0.650

Notes: The variables’ abbreviations come from the definitions and sources of the variables (Table 2).

**Table 7:** Multi-factor combination path analysis of high ACEE and low ACEE.

Configuration	High ACEE				Low ACEE	
	1	2	3		4	5
			3a	3b		
EAP	●	●	ⓧ	●	●	ⓧ
EGI	●	ⓧ	●	●	●	ⓧ
PC	ⓧ	ⓧ	ⓧ	●	●	ⓧ
FE	ⓧ	●	ⓧ	ⓧ		ⓧ
SP	ⓧ	ⓧ	●	●	ⓧ	ⓧ
Consistency	0.8473	0.8808	0.8889	0.8562	0.8814	0.8371
Raw coverage	0.0745	0.0893	0.1021	0.1719	0.1377	0.3435
Unique coverage	0.0329	0.0591	0.0416	0.1189	0.1020	0.3077
Overall solution consistency	0.9002				0.8444	
Overall solution coverage	0.3150				0.4455	

Notes: ● indicates the conditional variable’s presence; ⓧ indicates the conditional variable’s absence. A large icon represents a core element, and a small represents a peripheral element. The variables’ abbreviations come from the definitions and sources of the variables (Table 2).

## Data Availability

Not applicable.

## Appendix A

**Table A1:** The analysis of intermediate solutions and parsimonious solutions for high ACEE.

Configuration		Consistency	Raw Coverage	Unique Coverage	Solution Consistency	Solution Coverage
Intermediate solution	EAP*EGI*~PC*~FE*~SP	0.8473	0.0745	0.0329	0.9002	0.3150
	EAP*~EGI*~PC*FE*~SP	0.8808	0.0893	0.0591		
	~EAP*EGI*~PC*~FE*SP	0.8889	0.1021	0.0416		
	EAP*EGI*PC*~FE*SP	0.8562	0.1719	0.1189		
Parsimonious solution	EGI*~PC	0.8822	0.2062	0.0302	0.8485	0.3761
	EGI*~FE*SP	0.8881	0.2505	0.1048		
	EAP*~PC*FE	0.9286	0.1572	0.0000		
	EAP*~EGI*FE	0.7061	0.1242	0.0040		

**Table A2:** The analysis of intermediate solutions and parsimonious solutions for low ACEE.

Configuration		Consistency	Raw Coverage	Unique Coverage	Solution Consistency	Solution Coverage
Intermediate solution	EAP*EGI*PC*~SP	0.8814	0.1377	0.1020	0.8444	0.4455
	~EAP*~EGI*~PC*~FE*~SP	0.8371	0.3435	0.3077		
Parsimonious solution	EAP*PC*~SP	0.8886	0.1953	0.0987	0.8009	0.5030
	~EAP*~FE*~SP	0.8479	0.3799	0.0000		
	~EAP*~EGI*~FE	0.7923	0.3964	0.0245		
